# Development, Nutritional, and Sensory Characterisation of Whey–Hemp Hybrid High-Protein Bars

**DOI:** 10.3390/foods15213863

**Published:** 2026-10-28

**Authors:** Martin Pratto-Burgos, Miriam Ortega-Heras, María Dolores del Castillo, Inmaculada Gómez, Sonia Cozzano

**Affiliations:** 1 Departamento de Biotecnología y Ciencia de los Alimentos, Universidad de Burgos, Plaza Misael Bañueloss s/n, 09001 Burgos, Spain; mpb1023@alu.ubu.es; 2 Departamento de Biosistemas, Universidad Católica del Uruguay, Av. 8 de Octubre 2738, 11600 Montevideo, Uruguay; scozzano@ucu.edu.uy; 3 Instituto de Investigación en Ciencias de la Alimentación (CIAL, CSIC-UAM), C/Nicolás Cabrera, 9. Campus de la Universidad Autónoma de Madrid, 28049 Madrid, Spain; mdolores.delcastillo@csic.es

**Keywords:** hempseeds, whey protein, protein-rich food, plant-based protein alternatives, amino acid profile

## Abstract

This study evaluated the feasibility of developing hybrid high-protein bars through the partial replacement of whey protein with hempseeds. Three formulations with increasing hempseed-to-whey ratios (50/50, 60/40 and 75/25) were compared with a hempseed-free control. Proximate composition, dietary fibre, amino acid composition, and predicted protein-quality indices were determined. The fatty acid composition of the bars was estimated from the analysed ingredient profiles. Sensory characteristics were assessed using Flash Profile. Protein content decreased from 42.02 g/100 g in the control to 35.19 g/100 g in the 75/25 bar while meeting the “high protein” claim, with essential amino acids above the FAO adult reference pattern. Dietary fibre increased from 3.95 to 9.03 g/100 g and total fat from 25.97 to 33.78 g/100 g. The estimated proportion of saturated fatty acids decreased from 19.1% to 13.7%, whereas polyunsaturated fatty acids increased from 23.3% to 54.7%. Sensory analysis differentiated the formulations: the control was predominantly associated with chocolate-related attributes, whereas the hybrid bars showed more pronounced vegetal and textural attributes. The results support the feasibility of nutritionally differentiated high-protein bars by combining whey protein, hempseeds, and peanut butter, although changes should be interpreted as the outcome of the overall reformulation.

## 1. Introduction

In recent years, global demand for protein-rich foods has increased significantly, driven by growing consumer awareness of the role of dietary protein in maintaining health, enhancing physical performance, and promoting healthy ageing [1,2,3]. These trends are reflected in recent consumer surveys, which identify protein as one of the nutrients most actively sought by consumers and a key criterion when selecting healthy foods [3].

Current recommendations suggest a daily protein intake of 0.8 g kg^−1^ body weight for healthy adults, increasing to 1.4–2.4 g kg^−1^ for physically active individuals [4,5,6]. Adequate intake of high-quality protein also plays a crucial role in maintaining muscle health during ageing [2].

Whey protein is widely recognised as one of the highest-quality dietary protein sources due to high biological value, excellent digestibility, balanced essential amino acid profile, antioxidant activity, and desirable techno-functional properties, including emulsification [7,8,9,10,11,12,13]. These properties have led to the widespread use of whey protein in beverages and protein bars.

Although excessive whey protein intake has been associated with potential liver and kidney effects, increasing dietary fibre intake may help mitigate these risks [14,15,16,17]. Therefore, plant-based products have been increasingly incorporated into food formulations to enhance sustainability while complementing the nutritional properties of animal proteins.

In this line, hempseed (*Cannabis sativa* L.) is particularly attractive because it contains approximately 25% protein, 25% lipids, and considerable dietary fibre [18] while exhibiting low tetrahydrocannabinol content and a history of safe consumption [19,20]. Hemp proteins provide all essential amino acids, high bioaccessibility, elevated arginine content, favourable functional properties, and a lipid profile characterised by a low ω-6/ω-3 ratio [21,22,23,24]. Furthermore, Hemp provides a more favourable environmental footprint [25].

Hybrid protein foods combining animal and plant proteins have emerged as a promising reformulation strategy to improve nutritional quality, reduce animal protein content, and support more sustainable dietary patterns [26,27]. Consumer studies indicate that taste, healthiness, and convenience drive acceptance of these products [28].

In particular, whey-based hybrid formulations have been shown to benefit muscle protein synthesis, lean muscle mass, and physical performance [29,30]. Moreover, incorporating plant ingredients contributes dietary fibre, bioactive compounds, and improved sustainability without compromising the technological advantages of whey protein [17,31].

Previous studies have shown that replacing wheat flour with raw or roasted hemp flour in cookies significantly reduced hardness and increased the overall acceptability [32]. In gluten-free cakes, incorporating hemp flour at 25–100% increased protein content, although levels above 50% adversely affected physical and sensory properties [33]. Likewise, fortifying muffins with hempseed protein isolate increased protein content and antioxidant activity while preserving the product’s sensory characteristics [34].

These findings indicate that the incorporation of hempseed ingredients can modify the nutritional, technological, and sensory properties of different food matrices, with the magnitude of these changes depending on the ingredient and inclusion level [35,36]. However, limited information is available on the partial incorporation of hempseeds into whey-based high-protein bars and on the resulting balance between protein content, essential amino acid composition, dietary fibre, lipid composition, and sensory characteristics.

It was hypothesised that the overall reformulation involving the partial replacement of whey protein with increasing proportions of hempseeds would produce high-protein bars with an essential amino acid profile meeting the FAO reference pattern while increasing dietary fibre and modifying the estimated fatty acid composition and sensory characteristics. Therefore, the aim of this study was to evaluate the feasibility of developing hybrid high-protein bars through the partial replacement of whey protein with hempseeds and to determine whether the resulting formulations retained a high protein content and an essential amino acid profile meeting the FAO reference pattern.

Secondary objectives were to determine the proximate composition and dietary fibre content of the bars, estimate their fatty acid composition from the analysed ingredient profiles, and provide an initial characterisation of the sensory differences among formulations using Flash Profile.

## 2. Materials and Methods

### 2.1. Experimental Design

An iterative development framework was implemented, comprising three sequential phases: conceptualisation (opportunity identification), product development (prototyping), and screening-approached formulations based on the best-performing formulations [37].

**Figure 1:**
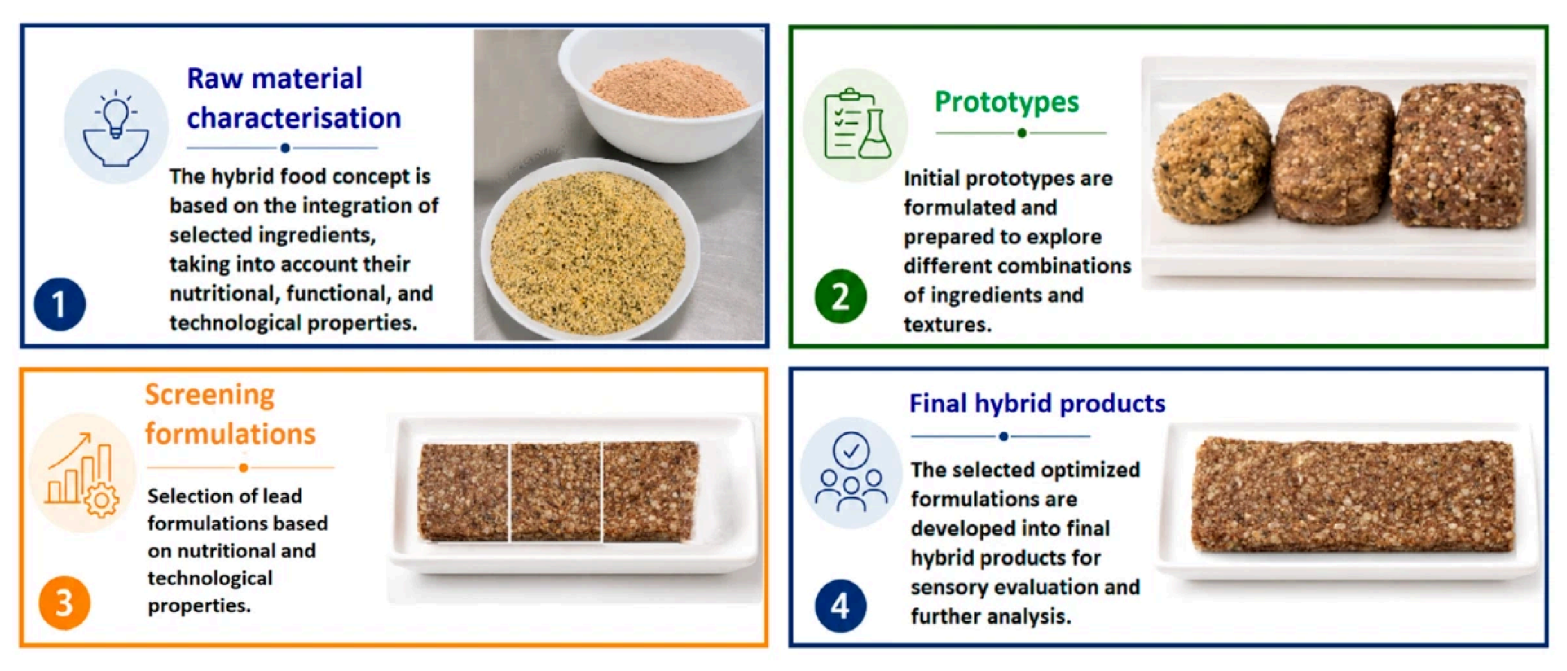
Graphical procedure for food design-thinking and product development.

Figure 1 illustrates the workflow followed for the development of the whey—hemp hybrid food based on hemp-derived ingredients and dairy components. The process began with the conceptualisation and design of the hybrid product, where different ingredient combinations were defined according to nutritional and technological objectives. Subsequently, prototypes were produced to evaluate the feasibility of the proposed formulations and to identify the most promising product structures and ingredient ratios.

#### 2.1.1. Conceptualisation of Whey–Hemp Hybrid Foods

The conceptualisation of whey–hemp hybrid foods was grounded in a complementary protein strategy aimed at integrating the technological functionality of dairy proteins with the sustainability and plant-based appeal of hemp. The hybrid food approach between whey and hemp was therefore designed to combine protein attributes within a single formulation. The protein attributes target improved nutritional quality, balanced amino acid composition, and techno-functional performance, achieving an optimal balance of physical attributes that may positively influence consumer acceptance [38].

From a formulation perspective, the conceptual design involved defining the relative proportion of whey and hemp based on three criteria: (i) nutritional complementarity (protein and fibre balance), (ii) functional compatibility within the intended food matrix, and (iii) sensory neutrality to minimise beany or dairy off-flavours. The hybrid food matrix was designed as a protein reformulation system rather than a simple blend to promote the desired nutritional and formulation feasibility. The development process was oriented toward the formulation of a hybrid whey–hemp system, conceived not merely as a mixture of ingredients but as a deliberately structured protein matrix.

Whey—hemp hybridisation was positioned as a transitional strategy between fully animal-based and fully plant-based formulations, potentially reducing environmental impact while maintaining familiarity for consumers accustomed to dairy proteins. This conceptual approach guided the subsequent selection of raw materials, formulation ratios, and processing parameters described in the following methodological section.

#### 2.1.2. Product Development

The whey—hemp hybrid protein bars (WHPBs) were elaborated with the following ingredients: whey protein, partially husked hempseeds, peanut butter, and water. The ingredients were purchased in a local market (Hipercor, Burgos, Spain). The chocolate-flavoured whey protein (Quamtrax, Madrid, Spain ) contained in 100 g (362 kcal): 81 g of protein; 5.4 g of carbohydrates; 1.4 g of fats, and 0.53 g of salt. The hempseeds (Natur^®^Green, Laboratorios Almond S.L, Murcia, Spain) contained in 100 g (580 kcal): 25 g of protein; 8.2 g of carbohydrates; 48 g of total fat; 0.10 g of salt; and 7.4 g of fibre. The peanut butter (Capitan Maní, Grupo Varma, Madrid, Spain) contained in 100 g (587 kcal): 24.35 g of protein; 21.26 g of carbohydrates; 49.66 g of total fat; and 8.4 g of fibre. These data were obtained from those shown on the labels of the products.

A preliminary formulation screening was conducted to identify the most suitable whey—hemp ratio in a reformulation of the whey-based system. As part of the product development process, several prototypes were iteratively prepared and qualitatively assessed for key technological and sensory attributes until a suitable consistency for human consumption was achieved. These evaluations were performed as initial formulation adjustments ensuring the feasibility of the different formulations.

#### 2.1.3. Screening-Approach Formulation

The formulations were developed through a preliminary screening of ingredients capable of binding the dry protein-rich components and providing sufficient cohesiveness for bar formation and handling. Date paste, carob, and peanut butter were evaluated as potential matrix-forming ingredients. Under the conditions tested, peanut butter provided the most suitable consistency, cohesiveness, and handling properties and was therefore selected for the final formulations. In addition to its technological function, its contribution to the protein and lipid composition of the bars was considered when interpreting the nutritional results.

The control C bar (C) was formulated with no hempseed addition, while three WHPBs were prepared with different portion percentages between ingredients, with the hempseeds/whey ratio exhibiting the widest compositional range: 50/50, 60/40, and 75/25 bars (Table 1).

With respect to the C bar, the optimal quantity of whey protein was based on practical experience to obtain a uniform consistency of the bar. Since whey has a fine powdered texture, it is more commonly incorporated into beverages than into solid foods. Consequently, it was necessary to assess different amounts of whey to determine the best blend and keep the bars from breaking apart.

WHPBs were prepared by first mixing whey protein with water until a homogeneous mousse was obtained. Hempseeds and peanut butter were then blended separately and incorporated into the whey mixture. The resulting dough was manually kneaded until homogeneous, compressed into a rectangular mould to achieve uniform thickness, and cut into bars of identical dimensions. The measures, in cm, of the bars were (length × width × height): 12.63 ± 0.25 × 3.40 ± 0.27 × 0.63 ± 0.04, and the weight, in g, was 42.95 ± 5.01.

**Figure 2:**
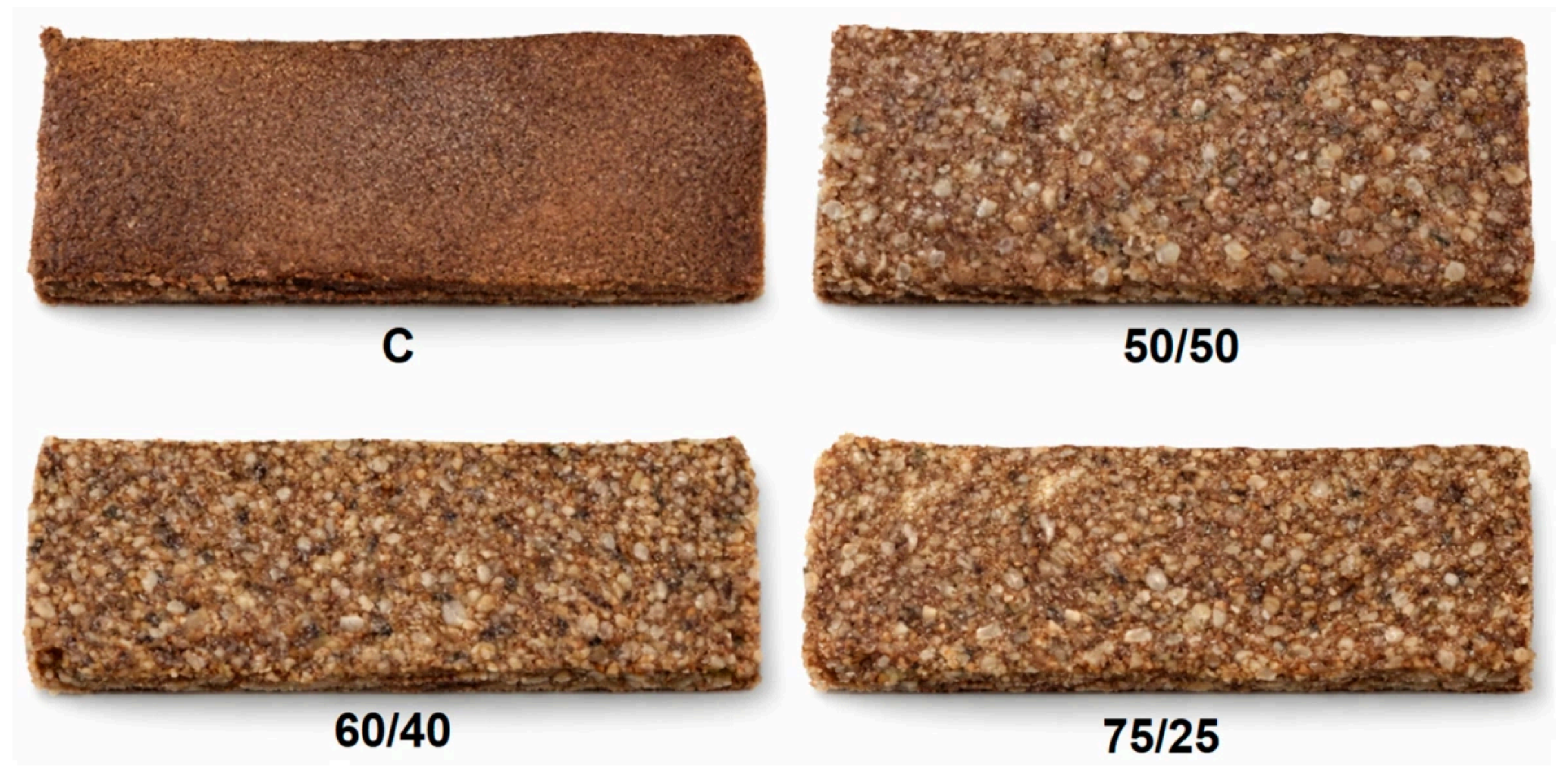
Whey—hemp hybrid protein bars and Control bar.

After moulding, the bars were refrigerated at 4 °C for 48 h to allow structure stabilisation before analysis (Figure 2). For each formulation, two independent batches were prepared 96 h apart. From each batch, three individual bars were sampled for each formulation. They were subjected to two repeated technical measurements. Thus, each formulation comprised two experimental batches, three bars per batch, and two technical measurements per bar (12 measurements in total for each WHPB and the C bar).

### 2.2. Proximate Composition and Physicochemical Analysis

Moisture content was determined by the difference in the sample weight before and after drying the sample at 105 ± 1 °C to constant weight, adapted from the AOAC method 930.15 [39]. Ash content was determined by incinerating the samples in a muffle furnace at 525 ± 1 °C (J.P. Selecta, Barcelona, Spain) for 5 hours, adapted from the AOAC method 923.03 [40]. Protein content was determined using the Kjeldahl method as described in AOAC method 2001.11 [41]. The nitrogen content was converted to protein using the factor 6.25. Fat content was determined by Soxhlet extraction according to AOAC method 920.39 [42] using petroleum ether in a Buchi B-811 extraction system (Büchi, Flawil, Switzerland). Total dietary fibre content was measured following the AOAC method 985.29 [43] using the enzyme kit TDF100A-1KT (Sigma-Aldrich^®^, St- Louis MO, USA). Carbohydrates were calculated by difference. For each formulation, all nutritional compositions were determined in duplicate for both batches.

The pH of the samples was determined using a calibrated digital pH meter 2001 (CRISON, Alella, Spain). Samples were grinded in a mortar, and 1 g was dispersed in 2 mL of distilled water.

Water activity (a_w_) was measured using a water activity meter (4TE AQUA LAB, Lleida, Spain). All measurements were performed in triplicate on three different bars for each formulation and for both batches to ensure accuracy and reproducibility.

### 2.3. Estimated Fatty Acid Profile Analysis

Lipid profile was determined separately for each raw material, since the gummy consistency of each bar and the presence of seed coats hindered efficient lipid extraction and could compromise the accurate lipid profile. Therefore, the lipid fraction of each ingredient was extracted and analysed individually, and the resulting values were subsequently weighted according to their proportion in each bar formulation (Table 1).

The lipid contribution of the whey protein was not determined separately because the commercial whey ingredient used in the formulations contained 1.4 g of fat in 100 g. This contribution was considered comparatively minor to the higher lipid content of the peanut butter and hempseeds (49.66 g/100 g and 48.00 g/100 g, respectively). Therefore, the estimated lipid profile of the final formulations was based on the lipid contribution of the ingredients providing the major lipid fraction.

Total lipids from samples were extracted and hydrolysed as described by Christie (2003) [44]. The extracted fatty acids were methylated, and the fatty acid methyl esters (FAMEs) were determined by gas chromatography using an Agilent 7890A Gas Chromatograph (Agilent Technologies Inc., Santa Clara, CA, USA) equipped with a flame ionisation detector (FID). A Fused Silica Capillary Column, Bond Cyanopropyl Silicone (60 m × 0.25 mm × 0.25 μm; Quadrex Corporation, Symta, Woodbridge, CT, USA) was employed. The injector and detector temperatures were set at 250 °C, and the injection volume was 1 μL in Split mode (100:1). Helium was used as the carrier gas at a constant flow rate of 1 mL/min. The initial oven temperature was 165 °C and was held for 10 min; it was then increased to 200 °C at a rate of 1.4 °C/min and held for 10 min. Finally, the temperature was raised from 200 °C to 210 °C at a rate of 2 °C/min and maintained at 210 °C for 5 min. FAMEs were identified by comparing peak retention times with those of standard compounds, and the relative areas were calculated considering the area of the internal standard C23:0. The results were expressed as the relative percentage of each fatty acid over the total identified fatty acids (SupelcoTM 37 FAME Mix 47885-U, Bellefonte, PA, USA). The lipid fraction of hempseeds and peanut butter was determined in triplicate.

### 2.4. Amino Acid Profile Analysis

Total amino acid composition was determined after acid hydrolysis of the samples, followed by ion-exchange chromatography. Briefly, samples were hydrolysed with 6 N hydrochloric acid under an inert atmosphere at 110 °C for 24 h to release protein-bound amino acids. Amino acid analysis was then performed using an automatic amino acid analyser Biochrom 30, based on ion-exchange chromatography followed by post-column derivatisation with ninhydrin. Norleucine was added to each sample as an internal standard.

Amino acid separation was carried out on an ion-exchange column using a gradient of lithium/sodium citrate buffers. After elution, amino acids continuously reacted with ninhydrin in a heated post-column reactor to form coloured derivatives. Detection was performed simultaneously by absorbance at two wavelengths: 570 nm, used as the main wavelength for the quantification of primary amino acids, and 440 nm, specifically used for the detection and quantification of proline.

Amino acids were identified by comparing their retention times with those of external amino acid standards. Quantification was performed by integration of the corresponding peak areas, and the values obtained were normalised using the recovery factor derived from the norleucine internal standard. Results were expressed as g amino acid per 100 g protein. Tryptophan was not determined because it is degraded under conventional acid hydrolysis conditions; therefore, tryptophan-dependent indices or interpretations should be considered with caution.

The predicted Protein Efficiency Ratio (PER) values were calculated from the amino acid composition of the samples using regression equations originally proposed to estimate in vivo protein quality from in vitro amino acid data. Three predictive indices, PER_1_ (Equation (1)), PER_2_ (Equation (2)), and PER_3_ (Equation (3)), were calculated according to the equations described by Alsmeyer et al. (1974) [45] and subsequently adopted in protein quality-evaluation studies, where amino acid contents are expressed as g per 100 g of protein.

(1) PER_1_ = −0.684 + 0.456 × Leu − 0.047 × Pro

(2) PER_2_ = −0.468 + 0.454 × Leu − 0.105 × Tyr

(3) PER_3_ = −1.816 + 0.435 × Met + 0.780 × Leu + 0.211 × His − 0.944 × Tyr

The Essential Amino Acid Index (% EAAi) was calculated as the geometric mean of the ratios between the concentrations of individual Essential Amino Acids (EAAs), represented by Equation (4), in the sample and their corresponding concentrations in the whole egg, represented by b, considered as the reference protein due to its high nutritional quality [46].

(4) %EAAi=100×Hisa ×Lysa ×Ilea ×Leua ×Meta ×Phea ×Thra ×Vala Hisb ×Lysb ×Ileb ×Leub ×Metb ×Pheb ×Thrb ×Valb n

Biological value (BV) was determined using the correlation proposed by Oser (1959) [46]:

(5) BV = EAAi × 1.09 − 11.7

The lysine/arginine (Lys/Arg) ratio was calculated as the quotient of lysine (Lys) and arginine (Arg) concentrations. Additionally, Branched-chain amino acids (BCAAs) were expressed as the sum of the branched-chain amino acids isoleucine (Ile), leucine (Leu), and valine (Val). The Fischer ratio, represented by Equation (6), was defined as the quotient of the sum of BCAAs and the sum of aromatic amino acids, namely tyrosine (Tyr) and phenylalanine (Phe).

(6) $$\mathrm{Fischer}=\frac{\mathrm{Ile}+\mathrm{Leu}+\mathrm{Val}}{\mathrm{Tyr}+\mathrm{Phe}}$$

### 2.5. Flash Profile

A Flash profile (FP) sensory analysis with experienced researchers (*n* = 21) of the Food Science and Technology Department of the Faculty of Science at Burgos University (Burgos, Spain) was done to generate a list of descriptive attributes of each formulation that, in their judgment, best discriminate among the WHPB’s samples and the C bar.

The FP method began with an introductory session in which the assessors were given an explanation about the procedure and were instructed to avoid the use of hedonic terms. After a short break, participants identified and selected sensory attributes by comparing the samples with one another. In the second session, each participant independently ranked the samples according to the perceived intensity of each attribute, scoring simultaneously using the terms generated during the previous session. All samples were presented simultaneously, identified by randomly assigned three-digit codes and presented in a randomised order to minimise potential order effects [47]. The samples measured 20 × 20 × 13 mm (length × width × height).

Attribute weighting techniques enable the discrimination of information among samples by identifying the variables that contribute most to sample differentiation [48]. This approach captures the diversity of the vocabulary used by each panelist and their perceptual discrimination.

All participants provided informed consent before participation. The study protocol was reviewed and approved by the Bioethics Committee of Burgos University (approval reference IO 12/2025, February 2025).

### 2.6. Statistical Analysis

Statistical differences among formulations, except for the estimated fatty acid profile and sensory analysis, were evaluated using one-way analysis of variance (ANOVA). Differences among formulations were assessed using Tukey’s significant difference post hoc test (*p* < 0.05).

Thus, each formulation comprised two batches; the resulting measurements were used to calculate the mean and standard deviation. Two-way ANOVA revealed no significant differences between the two batches (*p* > 0.1), indicating that the formulations were reproducible under the conditions evaluated.

All statistical analyses were conducted in R software, R Foundation for Statistical Computing (version 4.3.2, Vienna, Austria, 2023) [49], through the Posit Software RStudio integrated development environment (version 2022.12.0, Boston, MA, USA, 2022) [50].

Correlations between bars’ attributes gathered through FP were analysed by Generalised Procrustes Analysis (GPA). A consensus between attributes of each bar was established and graphically represented in 2D [51,52]. The shapes package [53], through the function procGPA, was used in the R environment. Additionally, the packages FactoMineR (v.2.13) [54] and Factoextra (v.2.0.0) [55] were applied.

## 3. Results and Discussion

### 3.1. Nutritional Content

Proximal composition data of the bars are shown in Table 2. As can be seen in this table, the moisture decrease is significant (*p* < 0.05) when the percentage of hempseeds, in the overall WHPBs reformulation, is above 50% (Table 2). A similar reduction in moisture with increasing seed content has been reported in chia-seed formulations [56]. However, its implications for the storage stability of the WHPBs would require direct shelf-life assessment.

Similar results were obtained for protein. It decreases significantly (*p* < 0.05) when the percentage of hempseeds increases in the overall WHPBs reformulation. This is explained by the high-protein contribution from whey products in relation to hempseeds (whey protein 81 g and hemp 25 g in 100 g). Hybrid products may exhibit reduced protein density due to ingredient substitution; plant-based incorporation can enhance dietary fibre content, enabling fibre-related nutritional claims [57]. Despite this reduction, all WHPBs retained a high protein content and met the conditions for a “high protein” nutrition claim under Regulation (EC) No 1924/2006.

As previously mentioned, WHPBs are hybrid products with whey protein, hempseeds, and peanut butter blends. The fatty acids present in the WHPBs were primarily attributable to hempseeds and peanut butter ingredients, as commercially available whey protein typically contains less than 2% total fat. A contrary effect is caused by the total fat content. The significantly (*p* < 0.05) highest total fat is when the percentage of hempseeds is high (Table 2). Hemp hearts have more than half of their nutritional value in total fat [23].

Ash content determines the minerals in any food product. The content of ashes increases when hempseeds are added to the overall WHPB reformulation (Table 2). This is according to Ambreen et al. (2023) [58] and Singh et al. (2020) [56], in which the authors’ research worked with different seed origins. The authors found that more seeds in the formulation lead to more ashes and higher mineral content. Ramos-Sanchez et al. (2025) [59] have shown that hemp seed-based foods and by-products are rich in micronutrients, including potassium, magnesium, and phosphorus.

Total dietary fibre content increases significantly (*p* < 0.05) between C bar and WHPB 50/50 and 60/40. It achieves the highest value at 75/25 WHPB. This could be explained by the characteristic of plant-based products of having high fibre content [60]. Particularly, hempseeds are considered a high source of fibre [22,61,62]. Although insoluble fibre was not determined in this study, hempseed dietary fibre has been reported to contain predominantly insoluble fractions, with arabinose and glucose as major components in the hemp seed hearts [59].

According to Regulation (EC) No 1924/2006 [63], all WHPB formulations met the conditions for the “high protein” nutrition claim, as protein provided approximately 30–36% of their total energy value, exceeding the minimum requirement of 20%. The 50/50 and 60/40 formulations met the conditions for the “source of fibre” claim, as they contained at least 3 g of dietary fibre per 100 g, whereas the 75/25 formulation met the conditions for the “high fibre” claim, with more than 6 g of dietary fibre per 100 g. As no sugars were added during formulation, the statement “contains naturally occurring sugars” may be used, provided that the composition of all commercial ingredients complies with the corresponding regulatory conditions.

The pH and a_w_ values are shown in Table 2. The pH increases significantly as 60% of hempseeds are added to the overall WHPB reformulation (from 6.17 ± 0.04, 60/40 bar, to 6.29 ± 0.04, 75/25 bar, *p* < 0.05). However, the implications of the observed pH differences for product stability cannot be established without direct shelf-life assessment.

In relation to a_w_, the value decreases with the addition of hempseeds. The minimum value corresponds to 75% of hemp, showing no significant difference (*p* > 0.05) with 60% of hempseeds’ addition. The lowest a_w_, in relation to C bar, is according to the research’s findings. AlJaloud et al. (2024) [64] studied whey protein and plant-based protein combination, which, at higher levels of plant-based protein, resulted in lower a_w_ values being reached. The a_w_ of WHPBs was between 0.848 and 0.871 (Table 2). Protein bars exceeding 0.65 a_w_ values had a significant impact on bar fragmentation [61].

After bar ingredients are mixed, water could migrate from those with high a_w_ to those with low a_w_ [65]. The decrease in a_w_ may be related to differences in water binding and redistribution among the components of the overall reformulated matrices [66,67]. However, the mechanisms responsible for this effect were not directly investigated in the present study.

### 3.2. Estimated Fatty Acid Profile

Lipids were the second-most abundant macronutrient fraction in the WHPBs, ranging from 26.0 to 33.8 g/100 g (Table 2). Furthermore, lipid content increased across the reformulated samples, and both hempseeds and peanut butter were major lipid-contributing ingredients. Therefore, estimation of the fatty acid profile was considered relevant for characterising the overall nutritional changes associated with the overall reformulation. Table 3 shows the fatty acid profile of the WHPBs, and the C bar was estimated based on the fatty acid content of the peanut butter and the hempseeds.

The estimated fatty acid composition differed among treatments, indicating that the overall reformulation modified the samples’ lipid profile. In general, changes were observed in the relative contribution of saturated, monounsaturated, and polyunsaturated fatty acids, reflecting the influence of the ingredients used in each formulation.

The estimated fatty acid composition differed among formulations, reflecting the contributions of hempseeds and peanut butter weighted according to their proportions in each formulation. Both ingredients are naturally rich in unsaturated fatty acids, although their relative contributions differ [22]. Across the reformulated bars, the estimated proportion of SFAs decreased from 19.1% in the hempseed-free control to 13.7% in the 75/25 WHPB, whereas the estimated proportion of PUFAs increased from 23.3% to 54.7% (Table 3).

The estimated proportion of MUFAs decreased from 56.3% in the control to 32.6% in the 75/25 formulation. MUFAs were the predominant fatty acid class in the control, whereas PUFAs represented the predominant class in the three WHPB formulations. The reduction in MUFAs was mainly associated with the lower relative contribution of cis-9-oleic acid, while the increase in PUFAs was mainly associated with higher relative contributions of linoleic and α-linolenic acids.

The estimated proportion of α-linolenic acid increased from 0.09% in the control to 9.93% in the 75/25 formulation (Table 3). This difference should be interpreted as the outcome of the overall reformulation rather than as the isolated effect of hempseeds, since the proportions of hempseeds, whey protein, peanut butter, and water varied among formulations.

Overall, the estimated fatty acid profile of the WHPBs showed lower relative SFA and MUFA proportions and higher PUFA proportions than the hempseed-free control. Estimated trans fatty acids also remained at trace levels in all formulations (<0.04%), in line with current recommendations to minimise trans fat intake [68]. However, the fatty acid composition of the finished bars was not directly measured, and the physiological relevance of these estimated differences requires experimental confirmation.

### 3.3. Amino Acid Profile Content

Whey protein provides higher protein quality, while plant proteins contribute fibre, healthy lipids, and bioactive compounds [69,70]. Due to its balanced, indispensable amino acid composition, whey protein is an effective complement to plant proteins with limiting amino acids, improving the nutritional quality of blended protein systems [71]. Consequently, hybrid protein foods have emerged as a promising reformulation strategy to combine the nutritional advantages of animal-and-plant-derived proteins while reducing dependence on animal protein sources and focusing on environmental sustainability [72].

The incorporation of plant-based protein into animal-based matrices modified the essential amino acid profile, although several indispensable amino acids remained relatively stable across formulations. Regarding essential amino acids, the 50/50 and 60/40 WHPBs maintained profiles comparable to the C bar, as no significant differences were observed for Valine (Val), Methionine (Met), Isoleucine (Ile), and Leucine (Leu) (*p* > 0.05). In contrast, the 75/25 WHPB showed significant reductions in Threonine (Thr), Phenylalanine (Phe), and Histidine (His), while Lysine (Lys) decreased significantly in all formulations compared with the C bar (Table 4).

When considered against the FAO adult maintenance amino acid scoring pattern [73], the reductions in Lys observed across the WHPBs, from 75.1 mg/g protein in the C bar to 73.6, 71.4, and 74 mg/g protein in 50/50, 60/40, and 75/25, respectively, are particularly relevant for protein quality assessment. However, despite the progressive decrease associated with hempseed incorporation, Lys concentrations remained above the FAO reference value for adults, indicating that the whey–hemp combinations maintained adequate Lys density relative to the adult reference pattern.

The lower Thr and His contents observed in the 75/25 WHPB (Table 4) suggest that increasing the hempseed proportion may progressively alter the amino acid profile toward that of plant-derived proteins; nonetheless, a favourable amino acid profile was maintained as all WHPBs exceeded the FAO-recommended reference values.

Additionally, the combined contribution of the sulphur-containing amino acids (Met + Cys) and aromatic amino acids (Phe + Tyr) also met the minimum requirement of the FAO, supporting the adequacy of the WHPBs in these essential amino acids. Conversely, no significant differences were detected for Ile, Leu, and Val across WHPBs and C bar. The overall reformulation of the whey-based system with increasing proportions of hempseeds suggests that it did not compromise these essential amino acids of the whey protein.

Considering the WHPB’s amino acids, the PER index is used to estimate protein quality based on the ability of a protein source to support growth (Table 4). Although three predicted PER values (PER_1_, PER_2_, and PER_3_) were calculated in this study based on different amino acid regression equations, the PER values discussed in previous studies correspond to the specific predictive model adopted by each author. Indices’ values over 2.7 indicate an excellent protein source, and when considering whey protein, the PER value increases to 3.2 [74]. Accordingly, high-protein products made with whey protein PER values are commonly lower than those of whey protein alone. It implies that those products have inferior protein quality, reflecting deficiencies in essential amino acid composition and/or reduced digestibility [74].

The predicted over-3.2 PER (PER_1_, PER_2_, PER_3_) values observed across the four samples, except for the 50/50 WHPBs, for PER_1_, indicate a high protein quality (Table 4). These results suggest that the amino acid composition of the bars is adequate to support protein utilisation. Although predicted PER values do not account for digestibility or bioavailability, they are widely accepted as useful indicators for the preliminary assessment of protein quality.

The percentage of EAAi observed in the WHPBs (Table 4) exceeded the percentage of EAA reported for plant-based foods (26% ± 2%) according to Gorissen et al. (2018) [75]. EAAi reflects the degree to which the essential amino acid profile matches a reference protein pattern [76,77]. The values obtained may be explained by the complementary amino acid contributions of whey protein and hempseeds. However, these predicted indices do not account for protein digestibility or amino acid bioavailability.

The BV, representing the absorbed dietary protein fraction retained for protein synthesis in the human body, indicates that the hybrid bars exhibited higher values than those from other plant-based sources: soy protein-74, pea protein-65, and rice protein-59 [77,78].

Compared with dairy proteins, the WHPBs exhibited a lower Lys/Arg ratio than milk (2.23) [79] and whey protein isolate (4.25) [80]. The lower Lys/Arg ratio observed in the WHPBs compared with the C bar was consistent with the amino acid profile of hempseed protein, in which lysine has been identified as a limiting indispensable amino acid, whereas arginine is present in relatively high proportions [81]. Thus, the progressive decrease in this ratio reflected the increasing contribution of hempseeds to the protein fraction of the WHPPBs.

Leucine and total BCAA contents did not differ significantly between most WHPBs and the C bar (Table 4). These results indicate that the overall reformulation did not substantially modify the relative contribution of BCAAs to the amino acid profile of the WHPBs.

The Fischer ratio obtained after the addition of hempseeds was lower than that reported for whey protein alone, approximately 3.73 [82], except for the 60/40 bar, which exceeded the value reported for whey protein (Table 4). However, the Fischer ratio was higher in the WHPBs than the values reported for conventional protein sources, including milk (1.95), egg (1.84), and soybean (1.64) [79]. The Fischer ratio differed among formulations, reflecting changes in the relative proportions of BCAA and aromatic amino acids.

The sulphur-containing amino acids Met and Cys are widely recognised as critical determinants of protein metabolic quality due to their roles in methylation reactions, glutathione synthesis, and overall protein turnover [83]. In contrast, many plant-based proteins are characterised by relatively low levels of sulphur amino acids, which may contribute to lower protein quality scores, for example, soy protein [76], when compared to protein sources with higher sulphur amino acid content [84]. In the overall WHPB reformulation, the partial replacement of whey protein with hempseeds does not affect Met and Cys concentrations; even the 75/25 bar showed the highest levels of Cys (Table 4).

Hemp has been proposed as a protein source of potential interest for the development of more sustainable food systems due to its relatively low agricultural input requirements when compared with animal-derived proteins [21,81,85]. However, the environmental performance of the ingredients and formulations was not assessed in the present study. Therefore, a comparative life-cycle assessment would be required to determine whether the partial replacement of whey protein with hempseeds provides an environmental advantage.

### 3.4. Flash Profile (FP)

The FP allows for describing the sensory characteristics of a product and identifying the differences and similarities between products. Each assessor created between 20 and 30 terms. From the 30 terms originally generated by the assessors, a final list of 22 terms was obtained (Supplementary Table S1).

A list was first compiled of all the terms generated by the judges; a synonym analysis was then carried out to group together those terms with similar meanings; furthermore, those terms that did not refer to sensory attributes (hedonic terms, emotion-related terms, etc.) were removed. Hedonic terms were removed from the dataset because the assessors had been instructed not to include such descriptors. This decision was consistent with the objective of the study, which was to generate a sensory characterisation of the products rather than to investigate affective responses. This stage was carried out by three researchers who initially worked alone to select valid terms and identify synonyms. They then pooled their results and reached a consensus.

**Figure 3:**
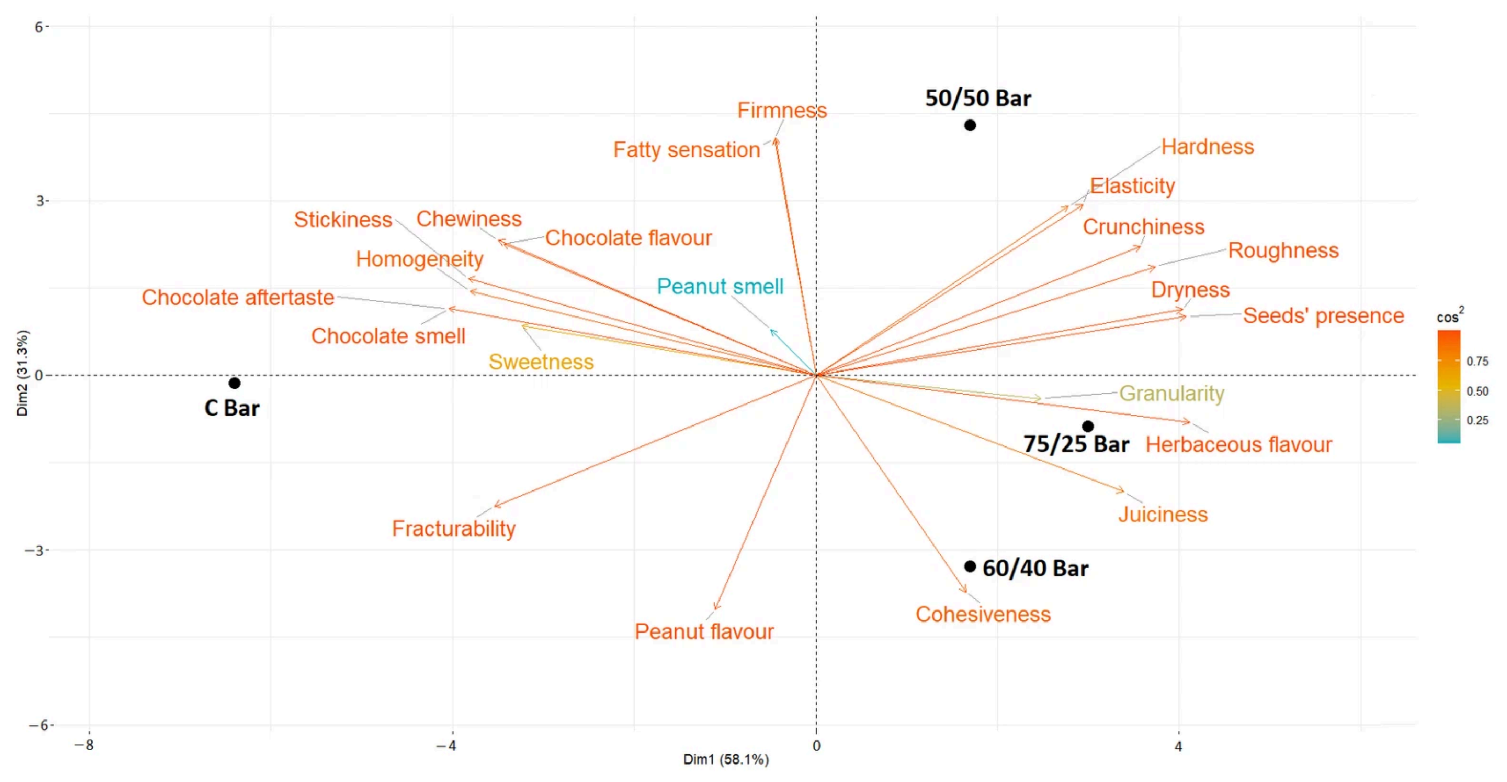
Biplot map of Generalised Procrustes Analysis performance based on Flash profile results. Variable importance on each axis is represented by cos2 (an indicator of variable quality representation; the closer to 1, the more the variable contributes to the corresponding axis).

Sweetness and stickiness were the attributes most frequently selected by participants (Supplementary Table S1). However, their overall frequency of use does not reveal how each attribute was perceived in individual samples, nor whether those perceptions differed between WHPBs and C bar. Therefore, GPA was applied, as it aligns individual assessors’ configurations into a consensus space, allowing sample differences to be identified while accounting for variations in how participants use and interpret attributes (Figure 3). The FP analysis revealed a clear sensory differentiation among the WHPBs, as represented by the first two dimensions, which accounted for 89.4% of the overall variation.

The opposite location of the C bar and the WHPBs plot (Figure 3) suggests that the reformulation induced substantial changes in the attributes evaluated. Samples positioned in opposite regions of the multivariate space are characterised by contrasting variable patterns [52], indicating that the reformulated products exhibit a distinct compositional and/or sensory profile compared with the conventional control. Such separation confirms that the introduced ingredients were major drivers of the observed variability.

The C bar was positioned on the negative side of dimension one and was mainly associated with chocolate-related attributes, including chocolate smell, chocolate flavour, chocolate aftertaste, sweetness, and texture-related attributes such as stickiness, chewiness, and homogeneity. These attributes indicate a more cohesive and confectionery-like sensory profile. In contrast, WHPBs were distributed on the positive side of dimension one, reflecting a progressive shift toward textural complexity and plant-derived sensory notes.

The 50/50 bar was strongly associated with firmness, fatty sensation, hardness, elasticity, crunchiness, roughness, dryness, and seed presence, suggesting a structurally compact and mechanically resistant matrix. The 60/40 bar was primarily characterised by cohesiveness and peanut flavour, indicating greater internal binding and a more pronounced peanut-related flavour perception.

Meanwhile, the 75/25 bar was linked to herbaceous flavour and juiciness, reflecting the greater contribution of plant-based ingredients and particulate perception. The higher pH values in the WHPBs (Table 2) may have influenced the overall flavour balance of the bars, potentially enhancing the perception of herbal notes associated with hemp-derived compounds. Additionally, the decrease in a_w_ in the WHPBs may have reduced water availability within the bar matrix, contributing to the hardness and dryness perceptions by participants.

Because the FP panel comprised experienced assessors rather than consumers, the resulting sensory map should be interpreted as a characterisation of perceived sensory differences among formulations rather than as an indicator of consumer liking, preference, or acceptance. The FP results therefore provide descriptive information on the sensory attributes that differentiated the formulations under the conditions of the present study. Overall, the sensory map demonstrates that increasing the plant—protein proportion progressively modifies both texture and flavour perception, shifting the products from a chocolate-dominant profile toward more complex structural and vegetal sensory characteristics.

## 4. Conclusions

The overall reformulation of the whey-based control with increasing proportions of hempseeds resulted in significant changes in the nutritional composition and sensory characteristics of the bars. Protein content decreased from 42.02 g/100 g in the hempseed-free control to 35.19 g/100 g in the 75/25 formulation. Nevertheless, all hybrid formulations met the conditions for the “high protein” nutrition claim, and their essential amino acid profiles remained above the FAO adult reference pattern.

Dietary fibre increased from 3.95 g/100 g in the hempseed-free control to 9.03 g/100 g in the 75/25 formulation, while total fat increased from 25.97 to 33.78 g/100 g. The estimated fatty acid composition also changed across formulations, with the relative proportion of SFAs decreasing from 19.1% to 13.7% and that of PUFAs increasing from 23.3% to 54.7%. As the fatty acid composition of the finished bars was estimated from the analysed ingredient profiles rather than measured directly, these results require confirmation by direct lipid analysis.

The sensory analysis differentiated the WHPBs from the hempseed-free control. The hempseed-free control was predominantly associated with chocolate-related attributes, whereas the reformulated bars were associated with more pronounced textural and vegetal attributes. As consumer acceptance was not evaluated, these sensory differences should not be interpreted as indicating preference or improved sensory quality.

Overall, the results demonstrate the feasibility of producing hybrid high-protein bars combining whey protein, hempseeds, and peanut butter. This approach resulted in a more compositionally diverse food matrix, although with lower protein content than the whey-based control. The findings should be interpreted as the outcome of the overall reformulation rather than as the isolated effect of hempseeds. Further studies should include direct fatty acid characterisation, protein digestibility, storage stability, consumer acceptance, and comparative environmental assessment.

This study is based on compositional analysis and predictive indices; it is not possible to conclude functional efficacy or clinical validation. Nevertheless, the conceptual and design-oriented framework established in this study provides a foundation for future empirical work and supports the strategic integration of hemp within animal protein matrices as a promising pathway for sustainability-oriented food innovation.

## Figures and Tables

**Table 1:** Ingredients of whey—hemp hybrid protein bars (50/50, 60/40, and 75/25 bars) and the C bar.

Ingredient	C	50/50	60/40	75/25
Whey protein product (g)	19.0	15.0	11.8	7.2
Hempseeds (g)	—	15.0	17.5	22.0
Peanut butter (g)	29.4	15.0	14.8	13.6
Water (mL)	12.5	9.8	8.5	7.5

Whey—hemp hybrid protein bar percentage relation corresponds to: 50:50 between hempseeds and whey (50/50 bar), 60:40 between hempseeds and whey (60/40 bar), and 75:25 between hempseeds and whey (75/25 bar). Values are expressed as g per formulation.

**Table 2:** Nutritional composition (g/100 g of weight) and physicochemical properties (pH and aw) of each whey—hemp hybrid protein bar and C bar.

	C	50/50	60/40	75/25
Moisture	16.41 ± 0.51 ^a^	15.22 ± 0.32 ^b^	12.83 ± 0.38 ^c^	11.71 ± 0.42 ^d^
Protein	42.02 ± 1.40 ^a^	40.97 ± 0.65 ^a^	38.45 ± 0.60 ^b^	35.19 ± 1.01 ^c^
Total fat	25.97 ± 0.68 ^d^	28.80 ± 0.47 ^c^	30.96 ± 0.88 ^b^	33.78 ± 0.71 ^a^
Carbohydrate	11.17 ± 0.22 ^a^	6.90 ± 0.55 ^b^	8.16 ± 0.72 ^b^	7.31 ± 0.14 ^b^
Ash	2.57 ± 0.07 ^b^	3.10 ± 0.04 ^a^	3.40 ± 0.08 ^a^	3.48 ± 0.05 ^a^
Total fibre	3.95 ± 0.31 ^c^	5.17 ± 0.53 ^b^	5.86 ± 0.61 ^b^	9.03 ± 0.78 ^a^
Energy (Kcal)	446 ± 13 ^b^	451 ± 9 ^b^	465 ± 13 ^a^	474 ± 11 ^a^
pH	6.12 ± 0.05 ^c^	6.19 ± 0.05 ^b^	6.17 ± 0.04 ^b^	6.29 ± 0.04 ^a^
a_w_	0.883 ± 0.007 ^a^	0.871 ± 0.004 ^b^	0.851 ± 0.004 ^c^	0.848 ± 0.009 ^c^

Whey—hemp hybrid protein bar percentages correspond to: 50:50 between hempseeds and whey (50/50 bar), 60:40 between hempseeds and whey (60/40 bar), and 75:25 between hempseeds and whey (75/25 bar). The control bar (C) without hempseeds addition. Data are presented as mean ± SD. Different superscript letters in the same row indicate statistically significant differences (p < 0.05). Carbohydrates were calculated by difference.

**Table 3:** Whey—hemp hybrid protein bars’ and control bars’ estimated lipid profile percentages.

Fatty Acid	C	50/50	60/40	75/25
Saturated fatty acids				
C16:0–Palmitic	9.56 ± 0.01	8.04 ± 0.20	7.90 ± 0.21	7.66 ± 0.21
C18:0–Stearic	3.27 ± 0.15	3.14 ± 0.11	3.12 ± 0.10	3.10 ± 0.09
C20:0–Arachidic	1.59 ± 0.12	1.10 ± 0.00	1.06 ± 0.00	1.00 ± 0.00
C22:0–Behenic	3.19 ± 0.01	1.74 ± 0.01	1.62 ± 0.01	1.40 ± 0.01
C24:0–Lignoceric	1.53 ± 0.01	0.83 ± 0.01	0.77 ± 0.01	0.67 ± 0.01
Monounsaturated fatty acids				
C18:1–cis-9 oleic	56.3 ± 0.5	37.4 ± 2.3	35.7 ± 2.4	32.6 ± 2.4
Omega-3 fatty acids				
C18:3–alpha-linolenic	0.09 ± 0.00	8.06 ± 0.04	8.73 ± 0.03	9.93 ± 0.03
C22:6 - docosahexaenoic (DHA)	0.226 ± 0.004	0.115 ± 0.003	0.106 ± 0.003	0.089 ± 0.002
Omega-6 fatty acids				
C18:2–Linoleic	23.1 ± 1.0	38.7 ± 2.8	40.2 ± 2.8	42.7 ± 2.7
C18:3–Gamma-linolenic	-	0.243 ± 0.002	0.264 ± 0.002	0.301 ± 0.001
ΣSFAs	19.1 ± 0.0	14.8 ± 0.0	14.4 ± 0.0	13.7 ± 0.0
ΣMUFAs	56.3 ± 0.5	37.4 ± 2.3	35.7 ± 2.4	32.6 ± 2.4
ΣPUFAs	23.3 ± 1.2	48.8 ± 0.1	50.9 ± 0.1	54.7 ± 0.1

Data are presented as mean ± SD. Values are estimated from the fatty acid profiles of hempseeds and peanut butter (*n* = 3) weighted by their proportion in each formulation; SD reflects the variability of ingredient analyses. Whey—hemp hybrid protein bar percentage relation corresponds to: 50:50 between hempseeds and whey (50/50 bar), 60:40 between hempseeds and whey (60/40 bar), and 75:25 between hempseeds and whey (75/25 bar). The control bar (C) without hempseeds addition. Trace amounts of the following fatty acids were detected: myristic, pentadecanoic, heptadecanoic, heneicosanoic, palmitoleic, cis-10-heptadecenoic, erucic, cis-11,14-eicosadienoic, nervonic, and trans-9-elaidic. ΣSFAs, total saturated fatty acids; ΣMUFAs, total monounsaturated fatty acids; ΣPUFAs, total polyunsaturated fatty acids.

**Table 4:** Amino acid composition (mg/g protein) of the whey—hemp hybrid protein bars and the control bar, and predicted protein-quality parameters (g/100 g amino acids).

**Amino Acid** **(mg/g Protein)**	**C**	**50/50**	**60/40**	**75/25**
Asp	120 ± 14 ^a^	126 ± 2.9 ^a^	127 ± 4 ^a^	131 ± 9 ^a^
Thr	76.0 ± 4.4 ^a^	74.2 ± 0.6 ^a^	72.8 ± 1.5 ^a^	56.9 ± 2.6 ^b^
Ser	81.6 ± 8.4 ^a^	81.6 ± 1.3 ^a^	40.1 ± 1.8 ^b^	44.9 ± 5.0 ^b^
Glu	270 ± 20 ^a^	244 ± 8 ^b^	251 ± 23 ^b^	229 ± 18 ^c^
Pro	74.3 ± 4.3 ^a^	78.4 ± 0.8 ^a^	50.9 ± 2.2 ^b^	74.0 ± 1.1 ^a^
Gly	44.4 ± 1.7 ^b^	46.2 ± 0.9 ^b^	57.4 ± 3.3 ^a^	46.6 ± 4.4 ^b^
Ala	58.9 ± 0.3 ^b^	56.2 ± 5.3 ^b^	43.0 ± 1.2 ^c^	66.6 ± 4.3 ^a^
Cys	9.7 ± 0.8 ^b^	9.6 ± 0.7 ^b^	9.4 ± 0.6 ^b^	12.9 ± 0.2 ^a^
Val	62.1 ± 4.3 ^a^	63.4 ± 2.1 ^a^	63.8 ± 2.0 ^a^	64.8 ± 1.7 ^a^
Met	13.6 ± 0.3 ^a^	14.9 ± 1.1 ^a^	12.4 ± 1.3 ^a^	13.7 ± 0.1 ^a^
Ile	73.1 ± 0.1 ^a^	72.9 ± 3.5 ^a^	73.9 ± 7.7 ^a^	69.2 ± 0.6 ^a^
Leu	138 ± 9.1 ^a^	149 ± 3.0 ^a^	143 ± 14.3 ^a^	114 ± 2.9 ^a^
Tyr	25.2 ± 0.6 ^a^	23.9 ± 0.8 ^a^	25.0 ± 1.2 ^a^	25.4 ± 0.5 ^a^
Phe	51.2 ± 2.5 ^a^	59.4 ± 2.9 ^a^	59.7 ± 5.1 ^a^	46.6 ± 2.2 ^b^
His	24.2 ± 1.5 ^a^	25.0 ± 2.5 ^a^	29.8 ± 3.1 ^a^	22.7 ± 0.5 ^b^
Lys	75.1 ± 4.4 ^a^	73.6 ± 1.6 ^b^	71.4 ± 6.9 ^b^	74.0 ± 2.8 ^b^
Arg	66.0 ± 4.9 ^b^	126 ± 8 ^a^	131 ± 12 ^a^	75.5 ± 3.4 ^b^
ΣEAA	514 ± 12 ^a^	532 ± 3 ^a^	530 ± 21 ^a^	461 ± 6 ^b^
ΣNEAA	750 ± 27 ^a^	791 ± 13 ^a^	734 ± 24 ^a^	705 ± 22 ^a^
ΣEAA/ΣNEAA	0.68 ± 0.04 ^a^	0.67 ± 0.02 ^b^	0.72 ± 0.05 ^a^	0.66 ± 0.03 ^b^
**Parameter (g/100 g** **Amino Acids)**	**C**	**50/50**	**60/40**	**75/25**
PER_1_	3.51 ± 0.06 ^a^	3.10 ± 0.05 ^b^	3.48 ± 0.30 ^a^	3.36 ± 0.09 ^a^
PER_2_	3.76 ± 0.07 ^a^	3.33 ± 0.05 ^b^	3.73 ± 0.34 ^a^	3.63 ± 0.10 ^a^
PER_3_	4.79 ± 0.11 ^a^	4.07 ± 0.10 ^a^	4.74 ± 0.66 ^a^	4.58 ± 0.08 ^a^
% EAAi	83.68 ± 1.28 ^a^	79.62 ± 0.88 ^b^	82.94 ± 2.60 ^a^	78.80 ± 0.89 ^b^
BV	79.51 ± 1.39 ^a^	75.09 ± 0.96 ^b^	78.71 ± 2.83 ^a^	74.20 ± 0.97 ^b^
Lys/Arg ratio	1.66 ± 0.06 ^a^	0.67 ± 0.05 ^b^	0.63 ± 0.08 ^b^	0.84 ± 0.07 ^b^
BCAA	21.79 ± 0.29 ^a^	20.03 ± 0.19 ^b^	21.65 ± 1.26 ^a^	20.85 ± 0.37 ^a^
Fisher ratio	3.75 ± 0.03 ^a^	3.08 ± 0.04 ^b^	3.83 ± 0.02 ^a^	3.33 ± 0.11 ^c^

Means ± SD; different superscript letters in the same row indicate statistically significant differences (*p* < 0.05). Abbreviations: EAA, essential amino acids; NEAA, nonessential amino acids; % EAAi, essential amino acid index; BCAA, branched-chain amino acids; BV, biological value; PER, protein efficiency ratio. Amino acid references: Asp, aspartic acid; Thr, threonine; Ser, serine; Glu, glutamic acid; Pro, proline; Gly, glycine; Ala, alanine; Cys, cysteine; Val, valine; Met, methionine; Ile, isoleucine; Leu, leucine; Tyr, tyrosine; Phe, phenylalanine; His, histidine; Lys, lysine; Arg, arginine. ΣEAA (sum of essential amino acids) = Thr + Val + Met + Ile + Leu + Phe + His + Lys. ΣNEAA (sum of non-essential amino acids) = Asp + Ser + Glu + Pro + Gly + Ala + Cys + Tyr + Arg. ΣEAA/ΣNEAA = ratio between the sum of essential and non-essential amino acids. ΣEAA was calculated excluding tryptophan, as this amino acid was not determined analytically; therefore, the reported % EAAi should be interpreted as predictive. CML, Nε-(carboxymethyl)lysine, was not reported. Trace amounts of GABA, γ-aminobutyric acid, were detected.

## Data Availability

All data supporting the findings of this study are publicly available in the Institutional Repository of the University of Burgos (RIUBU) and can be accessed at https://riubu.ubu.es/handle/10259/12288.

## Supplementary Materials

The following supporting information can be downloaded at: https://www.mdpi.com/article/10.3390/foods15213863/s1, Table S1: Frequency (N) and percentage (%) of the most commonly chosen attributes used by participants to evaluate the bars.

## Abbreviations

The following abbreviations are used in this manuscript:

WHPB	Whey—Hemp Hybrid Protein Bar
PER	Protein Efficiency Ratio
EAAi	Essential Amino Acid Index
BCAA	Branched-Chain Amino Acids
BV	Biological Value
SFAs	Saturated Fatty Acids
MUFAs	Mono-Unsaturated Fatty Acids
PUFAs	Poly-Unsaturated Fatty Acids.
