# Supplementary material for: Development, Nutritional, and Sensory Characterisation of Whey–Hemp Hybrid High-Protein Bars
Source: Foods. 2026 Oct 28;15(21):3863. doi: 10.3390/foods15213863 (PMC13763077; doi:10.3390/foods15213863)
Supplement: Supplementary file 1 [file foods-15-03863-s001.zip › foods-4573943-supplementary.pdf]

Supplementary table

Table S1. Frequency (N) and percentage (%) of mention of the attributes used by participants for the characterization of the bars. Percentages were calculated relative to the total number of participants (N = 21).

Attrribute	N	%
Sweetness	14	67
Stickiness	11	52
Hardness	6	29
Cohesiveness	6	29
Granularity	6	29
Homogeneity	5	24
Peanut flavour	5	24
Fatty sensation	3	14
Fracturability	3	14
Peanut smell	3	14
Chocolate flavour	3	14
Crunchiness	3	14
Chewiness	3	14
Roughness	2	10
Seeds' presence	2	10
Herbaceous flavour	2	10
Elasticity	2	10
Dryness	1	5
Chocolate smell	1	5
Firmness	1	5
Chocolate aftertaste	1	5
Juiciness	1	5
